# Successful aging defined by health-related quality of life and its determinants in community-dwelling elders

**DOI:** 10.1186/1471-2458-14-1013

**Published:** 2014-09-28

**Authors:** Chia-Ing Li, Chih-Hsueh Lin, Wen-Yuan Lin, Chiu-Shong Liu, Chin-Kai Chang, Nai-Hsin Meng, Yi-Dar Lee, Tsai-Chung Li, Cheng-Chieh Lin

**Affiliations:** Department of Medical Research, China Medical University Hospital, Taichung, Taiwan; School of Medicine, College of Medicine, China Medical University, Taichung, Taiwan; Department of Family Medicine, China Medical University Hospital, Taichung, Taiwan; Department of Physical Medicine and Rehabilitation, China Medical University Hospital, Taichung, Taiwan; Department of Psychiatry, Medical College, National Cheng-Kung University, Tainan, Taiwan; Medical Division, Sanofi Taiwan Co., Ltd, Taipei, Taiwan; Graduate Institute of Biostatistics, College of Management, China Medical University, Taichung, Taiwan; Department of Healthcare Administration, College of Medical and Health Science, Asia University, Taichung, Taiwan; China Medical University and Hospital, 2 Yude Road, Taichung, 40421 Taiwan

**Keywords:** Aged, Successful aging, Quality of life

## Abstract

**Background:**

Successful aging in old age is important. However, the determinants of successful aging vary across populations due to cultural differences, and only a limited number of studies have addressed these determinants in Taiwan population. This study aimed to evaluate successful aging via better physical and mental functions as well as to explore associated determinants in an elderly Taiwan population that had no impaired cognitive function.

**Methods:**

A community-based cross-sectional survey was conducted in January 2009 in Taichung, Taiwan. A total of 903 elderly persons (≥65 years) without impaired cognitive function were enrolled. Those with physical and mental component scores in the top tertile of the Short-Form 36 were considered to be aging successfully. All participants completed a structured questionnaire and the comprehensive geriatric assessment measurements of the five components of frailty defined by Fried et al. Crude and adjusted odds ratios (ORs) with 95% confidence intervals (CIs) were calculated to evaluate the relationship between associated factors and successful aging using logistic regression analysis.

**Results:**

The prevalence of successful aging was 10.4% in elders. A higher proportion of successful aging was found in non-frail (16.9%) and pre-frail elders (7.2%) than in frail elders (0.9%). Multivariate logistic regression showed pre-frail elders to be associated with lower prevalence of successful aging relative to non-frail elders (OR: 0.45; 95% CI: 0.24–0.84). Relative to those aged ≤70 years, elders aged 71–75 years were associated with a lower prevalence of successful aging (OR: 0.27; 95% CI: 0.13–0.58). Successful aging was also more likely among those able to visit relatives and friends (OR: 3.86, 95% CI: 1.09–13.61) and among those without a history of falling (OR: 4.95; 95% CI: 1.79–13.74), pain (OR: 4.04; 95% CI: 2.18–7.50), or sleep disorders (OR: 2.36; 95% CI: 1.30–4.27).

**Conclusion:**

Successful aging was associated with age, frail status, chronic health-related problems and psychosocial support. However, whether or not these associations are causal requires further exploration.

## Background

The proportion of the elderly population aged ≥65 years has continued to dramatically increase worldwide from 8% in 1950 to 11% in 2009 [1]. A similar tendency has occurred in Taiwan, as the number of elderly people increased by approximately 2% each year throughout the 1990s. The proportion of elderly Taiwanese is projected to further increase almost threefold from 10% in 2009 to 36% by 2050. Because the aging process is associated with increased susceptibility to chronic conditions, disabilities, psychosocial problems and comorbidities [2, 3], it is important that this aging population is able to enter late stages of life in relatively good health.

An extensive review of 28 quantitative studies reported that there are several methods to operationally define and measure successful aging [4]. The prevalence and definitions of successful aging were found to vary among studies [4]. As the number of included domains increased, definitions of successful aging tended to become more complex [5]; there was, however, a consensus regarding the multidimensional nature of successful aging [4–6]. The Study of Health Assessment and Risk in Ethnic groups also reported a cross-national variation in the prevalence of successful aging among 14 European countries. The authors suggested that self-reported health measures may be the most appropriate measures for comparing cross-national differences to avoid diagnostic differences across countries [7]. Multidimensional, standardized, and self-rated instruments are more practical and are more easily comparable among different countries and populations.

The Short Form-36 (SF-36) is a multidimensional scale and a well-established instrument that measures health concepts and self-reported health-related quality of life [8]. Using SF-36 to define successful aging is acceptable because it represents overall health status in two key measures—the physical component summary (PCS) and the mental component summary (MCS) scores. These two scores reflect the status of physical, mental, and social well-being that are generally used to define health status [9]. The aim of this study was to explore the prevalence of successful aging, as gauged by SF-36, as well as to identify determinants of successful aging among community-dwelling elders.

## Methods

### Population and participants

We conducted a population-based cross-sectional study in June 2009 with a target population of all residents aged ≥65 years residing in eight administrative neighborhoods of Taichung, Taiwan. Taichung is located in west-central Taiwan and has a population of just over one million people, making it the third largest city on the island; its area spans 163.4 km^2^, and the population density is 6,249/km^2^ (2009). There were 3,997 elderly residents in these eight administrative neighborhoods of Taichung, accounting for 4.5% of the population. Data for this study were obtained from individuals’ records compiled by the Bureau of Households; details of this study’s sampling method are described elsewhere [10]. This research was approved by the Human Research Committee of China Medical University Hospital, and written informed consent was obtained from each participant. A total of 1,347 elderly residents of Taichung participated in this study, yielding an overall response rate of 49.0%. Among these participants, 256 elderly residents completed only the first stage of the screening test for the frailty assessment and did not submit responses to the SF-36 questionnaire. A total of 903 elders were included in the final data analysis after excluding those diagnosed with dementia (n = 18), those lacking Mini-Mental State Examination (MMSE) information (n = 5), those with MMSE scores less than 14 points (n = 8), those who had incomplete frailty-related components (n = 87), and those with incomplete SF-36 information (n = 70).

### Measurements

#### Successful aging

Measurements from the SF-36 instrument were applied to identify whether the elderly residents of Taichung were aging successfully. The SF-36 is a short questionnaire with 36 items that measures the following eight multi-item variables: physical functioning (PF, 10 items), social functioning (SF, 2 items), role limitations due to physical problems (RP, 4 items), role limitations due to emotional problems (RE, 3 items), mental health (MH, 5 items), vitality (VT, 4 items), pain (BP, 2 items), and general perception of health (GH, 5 items). The scores for each variable item were coded, summed, and transformed into a score ranging from 0 (worst measured health state) to 100 (best measured health state). The SF-36 PCS and MCS scales were derived from the standard SF-36 scoring algorithms. Elders with both PCS and MCS in the highest tertile, indicating the best states of physical and mental health, were considered to be successfully aging. The cutoff scores of the top tertile were 53.00 for the PCS and 59.28 for the MCS.

##### Socioeconomic factors, health-related practices, visual and hearing capacity, and psychosocial support

Data on health-related practices, psychosocial support, and socioeconomic factors (including age, gender, education, marital status and money use) were collected through self-administered questionnaires. Visual and hearing capacities were categorized as good, general, and bad/blind or bad/deaf. Health-related practices, such as tobacco use and alcohol consumption, were categorized as never and former/current. Regular exercise was categorized as “yes” or “no”. Psychosocial support was assessed using two statements: “Someone is listening when you talk” and “You see relatives/friends when you want”.

##### Frailty status and chronic illness and problems

We adopted the definition of frailty proposed by Fried et al. [11], consisting of five components: unintended weight loss, weakness, poor endurance and energy, slowness, and low physical activity level. In this study, unintended weight loss was defined as weight loss ^3^3 kg over the course of the previous year; weakness was defined as grip strength in the lowest quintile at baseline, based on the subgroups of gender and body mass index. Poor endurance and energy were evaluated by self-reported exhaustion and by two questions from the Center for Epidemiological Studies-Depression scale [12]. Slowness was measured for each gender and height subgroup and was defined as the slowest quintile to walk a distance of 15 feet and was divided into gender and height subgroups [11]. Low physical activity level was measured for each gender and was defined as the lowest quintile of kilocalories spent every week (using weighted scores), based on responses from each participant. Subjects exhibiting none of the above components were considered robust; those with one or two components were considered pre-frail; and those with more than two components were considered frail.

Self-reported personal medical histories—including diabetes mellitus, hypertension, heart disease, stroke, arthritis, hyperlipidemia, gout, hyperuricemia, cataract, and fall histories—were collected. Pain problems and sleep disorders were also collected as “yes” or “no” binary responses.

### Statistical analysis

Continuous variables were reported as mean ± standard deviation (SD), and categorical variables were reported as numbers and percentages. Student’s *t*-test was used to compare the eight dimensions and the two component summaries of SF-36 among elders who were and were not aging successfully. Univariate logistic regression was used to explore the associations of sociodemographic factors, psychosocial support, visual and hearing capacities, health-related practices, and chronic health problems with successful aging. Variables found to be statistically significant by univariate logistic regression analysis were selected to further evaluate their relative contributions using four multivariate logistic regression models. First, the sociodemographic and psychosocial support factors were evaluated by multivariate logistic regression. Health-related practices and visual and hearing capacities were then added to the second model; chronic illness and fall history were then added to the third model; and pain, sleep disorders, and frailty were finally added to the fourth model. All calculations were repeated for sensitivity analysis. The cutoff point of both the PCS and the MCS for defining successful aging was changed from the highest tertile to the seventieth percentile and the fourth quartile. All reported p-values were those of two-sided tests; significance was defined as p < 0.05. All analyses were performed using SAS version 9.2 statistical software (SAS Institute Inc., Cary, NC, USA).

## Results

Of the 903 elders (mean age, 73.90 years) enrolled in this study, 10.4% were identified as successful agers. Successfully aging elders had significantly higher scores in eight SF-36 dimensions than those who were not successfully aging; they also had the most favorable health status (with scores of 100) in the RP, SF, and RE variables (Table 1).

**Table 1:** **Comparison of eight dimensions and two component summaries of SF-36 between successfully aging and not successfully aging elders**

	Not successfully aging		Successfully aging		
	(n = 809)		(n = 94)		
	Mean	SD	Mean	SD	p value
PF	80.99	20.14	96.17	4.49	<0.001
RP	83.87	34.17	100.00	0.00	<0.001
BP	78.57	21.31	98.45	5.72	<0.001
GH	59.41	19.60	84.85	10.05	<0.001
VT	70.90	18.55	93.99	6.54	<0.001
SF	91.12	16.27	100.00	0.00	<0.001
RE	90.40	27.80	100.00	0.00	<0.001
MH	79.18	16.18	97.36	3.29	<0.001
PCS	47.50	8.07	55.32	1.41	<0.001
MCS	54.61	7.68	61.31	1.11	<0.001

Physical functioning (PF), Role physical (RP), Bodily pain (BP), General health (GH), Vitality (VT), Social functioning (SF), Role emotional (RE), Mental health (MH), Physical component summary (PCS), and Mental component summary (MCS).

Older elders, females, and elders without enough or with just enough money for personal use had a lower prevalence of successful aging. The unadjusted analysis showed a higher prevalence of successful aging among elders with ≥7 years of education, with general or good visual capacity, with good hearing capacity, who regularly exercised, and who were able to see relatives and friends whenever they wished (Table 2).

**Table 2:** **Prevalence and odds ratio of successful aging defined by health-related quality of life among socio-demographic factors, visual and hearing capacity, and health-related practices**

Variable	Total n	Successful aging		OR	95% CI
		n	%		
Total	903	94	10.4	***-***	***-***
***Socio-demographic factors***					
Age (years)					
≤70	339	54	15.93	1.00	
71–75	226	14	6.19	**0.35**	**(0.19, 0.64)**
>75	338	26	7.69	**0.44**	**(0.27, 0.72)**
Gender					
Men	477	61	12.79	1.00	
Women	426	33	7.75	**0.57**	**(0.37, 0.89)**
Education					
Illiterate	103	3	2.91	1.00	
≤6 years	229	16	6.99	2.50	(0.71, 8.79)
7–12 years	308	42	13.64	**5.26**	**(1.60, 17.36)**
≥ 13 years	240	33	13.75	**5.31**	**(1.59, 17.75)**
Marital status					
Current	644	74	11.49	1.00	
Others	256	20	7.81	0.65	(0.39, 1.09)
Money use					
Enough	210	32	15.24	1.00	
Just enough	578	57	9.86	**0.61**	**(0.38, 0.97)**
Not enough	111	5	4.50	**0.26**	**(0.10, 0.69)**
***Visual and hearing capacity***					
Visual capacity					
Bad/blind	245	10	4.08	1.00	
General	410	42	10.24	**2.68**	**(1.32, 5.45)**
Good	245	42	17.14	**4.86**	**(2.38, 9.94)**
Hearing capacity					
Bad/deaf	152	9	5.92	1.00	
General	308	17	5.52	0.93	(0.40, 2.13)
Good	443	68	15.35	**2.88**	**(1.40, 5.93)**
***Health-related practices***					
Regular exercise					
No	217	11	5.07	1.00	
Yes	683	83	12.15	**2.59**	**(1.35, 4.96)**
Smoking					
No	711	74	10.41	1.00	
Current	82	12	14.63	1.48	(0.76, 2.85)
Former	110	8	7.27	0.68	(0.32, 1.44)
Drinking					
No	727	70	9.63	1.00	
Current	121	17	14.05	1.53	(0.87, 2.71)
Former	55	7	12.73	1.37	(0.60, 3.14)
***Psychosocial support***					
Someone is listening when you talk					
No	59	3	5.08	1.00	
Yes	842	91	10.81	2.26	(0.69, 7.37)
You see relatives/friends when you want					
No	114	3	2.63	1.00	
Yes	786	90	11.45	**4.78**	**(1.49, 15.38)**

Results in bold are significant at p<0.05.

Among the associations with chronic health problems, increased odds of successful aging were found in non-frail elders and in those without hypertension, heart disease, hyperuricemia, arthritis, cataracts, fall histories, pain problems, or sleep disorders (Table 3).

**Table 3:** **Prevalence and odds ratios of successful aging defined by health-related quality of life among chronic illness, problems, and frailty status**

Variable	Total n	Successful aging		OR	95% CI
		n	%		
Hypertension					
Yes	474	37	7.81	1.00	
No	420	57	13.57	**1.85**	**(1.2, 2.87)**
Diabetes mellitus					
Yes	154	12	7.79	1.00	
No	740	81	10.95	1.45	(0.77, 2.74)
Heart disease					
Yes	264	13	4.92	1.00	
No	639	81	12.68	**2.80**	**(1.53, 5.13)**
Hyperlipidemia					
Yes	226	21	9.29	1.00	
No	662	73	11.03	1.21	(0.73, 2.02)
Gout					
Yes	110	7	6.36	1.00	
No	781	87	11.14	1.84	(0.83, 4.09)
Hyperuricemia					
Yes	105	4	3.81	1.00	
No	786	89	11.32	**3.22**	**(1.16, 8.97)**
Arthritis					
Yes	178	7	3.93	1.00	
No	691	82	11.87	**3.29**	**(1.49, 7.25)**
Stroke					
Yes	48	0	0.00	1.00	
No	837	94	11.23	-	--
Cataract					
Yes	421	32	7.60	1.00	
No	477	62	13.00	**1.82**	**(1.16, 2.84)**
Fall history					
Yes	702	87	12.39	1.00	
No	188	7	3.72	**3.65**	**(1.66, 8.04)**
Pain problem					
Yes	471	19	4.03	1.00	
No	401	74	18.45	**5.38**	**(3.19, 9.09)**
Sleep disorder					
Yes	394	22	5.58	1.00	
No	497	71	14.29	**2.82**	**(1.71, 4.64)**
Frailty					
Non-frail	368	62	16.85	1.00	
Pre-fail	428	31	7.24	**0.39**	**(0.24, 0.61)**
Frail	107	1	0.93	**0.05**	**(0.01, 0.34)**

OR: odds ratio; 95% CI: 95% confidence interval; Results in bold are significant at p<0.05.

Nested logistic regression analysis was used to further examine the interrelatedness of factors significantly associated with successful aging. Among socioeconomic and psychosocial support factors, the odds of successful aging were significantly lower for the very elderly and for those with insufficient income. After accounting for the effects of visual capacity, hearing capacity, and exercise habits on successful aging, the significance of age remained unchanged while the strength of the financial resources association diminished. Meanwhile, we observed increased odds of successful aging for elders with general or good visual capacity and for those who exercised regularly. After chronic illness and history of falling were added into the regression model, successfulness of aging was statistically associated with an age range of 71–75 years; regular exercise; and the absence of heart disease, arthritis, and fall histories.

The associations of regular exercise and chronic diseases with successful aging disappeared, however, after adding the factors of pain, sleep disorders and frailty in the fourth model. In the final model, the odds of successful aging decreased in elders aged 71–75 years relative to those ≤70 years (adjusted odds ratio [OR]: 0.27; 95% confidence interval [CI]: 0.13–0.58) and in those in pre-frail states. An increase in the odds of successful aging was observed in elders who were able to visit relatives and friends whenever they wished (OR: 3.86; 95% CI: 1.09–13.61) and in those without a history of falling (OR: 4.95; 95% CI: 1.79–13.74), pain (OR: 4.04; 95% CI: 2.18–7.50), or sleep disorders (OR: 2.36; 95% CI: 1.30–4.27) (Table 4).

**Table 4:** **Nested logistic regression analysis of successful aging defined by health-related quality of life**

		Model I			Model II			Model III			Model IV		
Variable	Effect	OR	95% CI		OR	95% CI		OR	95% CI		OR	95% CI	
Age (years)	71–75 vs ≤70	**0.36**	**0.18**	**0.69**	**0.36**	**0.18**	**0.70**	**0.34**	**0.17**	**0.70**	**0.27**	**0.13**	**0.58**
	>75 vs ≤70	**0.38**	**0.22**	**0.67**	**0.41**	**0.23**	**0.74**	0.53	0.28	1.02	0.60	0.30	1.21
Gender	Women vs Men	0.64	0.39	1.08	0.79	0.46	1.36	0.88	0.50	1.56	1.08	0.58	2.00
Education	≤6 years vs Illiterate	1.49	0.41	5.41	1.07	0.28	4.03	0.99	0.26	3.88	1.03	0.25	4.27
	7–12 years vs Illiterate	2.87	0.84	9.79	1.91	0.54	6.75	1.93	0.53	7.03	1.61	0.42	6.22
	≥ 13 years vs Illiterate	2.86	0.81	10.11	2.18	0.60	7.99	2.18	0.58	8.28	1.87	0.46	7.63
Money use	Just enough vs Enough	0.76	0.45	1.27	0.85	0.50	1.45	0.90	0.51	1.60	1.04	0.56	1.93
	Not enough vs Enough	**0.35**	**0.12**	**0.97**	0.40	0.14	1.14	0.39	0.13	1.16	0.54	0.17	1.75
You see relatives/friends when you want	Yes vs No	3.08	0.93	10.22	2.77	0.82	9.36	3.45	1.00	11.89	**3.86**	**1.09**	**13.61**
Visual capacity	General vs Bad/Blind				**2.32**	**1.04**	**5.18**	2.07	0.90	4.73	1.36	0.57	3.27
	Good vs Bad/Blind				**2.15**	**1.01**	**4.60**	2.06	0.94	4.54	1.67	0.74	3.79
Hearing capacity	General vs Bad/Deaf				2.00	0.84	4.73	1.57	0.64	3.81	1.78	0.70	4.54
	Good vs Bad/Deaf				0.66	0.25	1.77	0.53	0.19	1.46	0.48	0.17	1.36
Regular exercise	Yes vs No				**2.71**	**1.25**	**5.87**	**2.73**	**1.24**	**6.02**	1.53	0.61	3.79
Hypertension	No vs Yes							1.42	0.84	2.42	1.38	0.78	2.42
Heart disease	No vs Yes							**2.07**	**1.01**	**4.26**	2.06	0.96	4.42
Hyperuricemia	No vs Yes							3.16	0.92	10.87	3.19	0.89	11.49
Arthritis	No vs Yes							**2.47**	**1.06**	**5.80**	1.82	0.74	4.45
Cataract	No vs Yes							1.05	0.59	1.86	1.07	0.59	1.97
Fall history	No vs Yes							**4.08**	**1.55**	**10.72**	**4.95**	**1.79**	**13.74**
Pain problem	No vs Yes										**4.04**	**2.18**	**7.50**
Sleep disorder	No vs Yes										**2.36**	**1.30**	**4.27**
Frailty	Pre-fail vs Non-frail										**0.45**	**0.24**	**0.84**
	Frail vs Non-frail										0.14	0.02	1.13
Adjusted McFadden’s R^2^		9.1%			16.0%			23.4%			32.3%		
Likelihood ratio test^†^		-			p < 0.001			p < 0.001			p < 0.001		

OR: odds ratio; 95% CI: 95% confidence interval; Results in bold are significant at p<0.05.

^†^: The current model compared with prior model.

The sensitivity analysis results showed that age, fall history, and pain were still significantly associated with successful aging when using the highest cutoff value at the seventieth percentile for both the PCS and the MCS. Successful aging associations with sleep disorders and visiting relatives and friends were strong but insignificant. Using the highest quartile as a cut-off value, only fall history and pain were significantly associated with successful aging. All elders who were frail were found to be unsuccessfully aging; however, the factor of frailty could not be added to the final multivariate model due to problems with statistical convergence.

## Discussion

The prevalence of successful aging, as was defined using SF-36, was 10.4% in community-dwelling elders in a metropolitan population of Taiwan. Elders with chronic diseases or other health-related problems had a lower prevalence of successful aging compared with those without such health issues. All elderly people who had had a stroke, for example, were considered to be unsuccessfully aging. Elders more likely to be successfully aging, however, were ≤70 years old; were are able to visit relatives or friends whenever they wished; and had no history of falling, pain, or sleep disorders.

We adopted the five components of frailty defined by Fried et al. [11] that included objective, performance-based measures. A previous study had reported that frailty in elders is an important clinical state that is distinct from normal aging and a strong predictor for disability [11]. In this study, frailty was found to be an independent determinant of successful aging. Among frail elders, only one (0.93%) was found to be successfully aging; and even after PCS and MCS cutoff values were increased from tertile to a higher percentile, no other frail elders were found to be successfully aging. These results indicated that gauging successful aging using SF-36 is not only easy but also reflects actual health conditions. A noteworthy additional study by Theou et al., however, reported that frailty in elders can be reversed by structured exercise training [13].

The prevalence of successful aging among previous community-based studies ranges from 0.4% to 95% based on various definitions [5, 6, 12, 14–20]. In general, those studies using a single-item, self-rating scale reported a higher and more variable prevalence of successful aging, ranging from 50.3% to 90.2% [6, 15, 18]. The focus of many studies on successful aging has shifted from disease status and functional decline to multidimensional health status, which encompasses physical, functional, psychological, and social health [21]. Early published reports defined successful aging using only one or two dimensions of health, such as the absence of chronic diseases, longer longevity, independent physical functioning, social life engagement, and mental health [14, 16, 17, 19]. However, more recent studies have focused on the concept that successful aging is more multifaceted and complex [5, 7]. Studies using the absence of major disease as one of the domains to define successful aging in elders reported a lower prevalence of successful aging [15, 18, 20] compared with those not using this domain [5, 7, 12, 15]. To obtain a comparable prevalence of successful aging among elders and to explore the associated determinants, there is a need for a simple, standardized and multidimensional tool to be used across studies. In a study conducted in Hong Kong, higher PCS and MCS scores in SF-36 reflected better overall physical, functional, psychological, and social health in Chinese elders [22]. Consequently, higher PCS and MCS scores in SF-36 among elders were used to define successful aging in this study.

Age is a commonly identified determinant of successful aging. Consistent with the results reported in previous studies [5, 23, 24], younger age was one determinant of successful aging among elders in this study. We also found that the ability to see relatives and friends at will (as a variable related to social support) was an independent determinant for successful aging among the elderly. Pruchno et al. [23] reported that unsuccessfully aging elders have less social support compared with successful agers. Our findings on the associations among sleep disorders, fall histories, and successful aging were similar to those in prior research [25]. Elderly persons with sleep disorders were at a higher risk of unsuccessfully aging compared with those without sleep disorders. This finding provides evidence supporting a previous study by Andrews et al. [25] that defined the concept of high functioning and found that elders without sufficient sleep were less likely to be in the high functioning category. In our findings, a history of falling was associated with a lower likelihood of successful aging, as those with such a history tend to restrict outdoor activity out of a fear of falling [26]. In general, the fear of falling has tended to impede elderly participation in healthy activities and leads to decreased mobility, resulting in diminished quality of life [27, 28].

Previous studies have reported that chronic diseases—such as arthritis, diabetes, stroke, and hypertension—were all associated with successful aging. [5, 16] We found that these diseases were not independent risk factors of unsuccessful aging because their effects were explained by falls, frailty, pain, and sleep problems. Among these chronic diseases, stroke had a major impact on successful aging; all elders who had a history of stroke in this study were found to be unsuccessfully aging. Strokes were not, however, added to the final multivariate model due to problems with statistical convergence. We observed lower physical component scores in elders with stroke histories compared with those without stroke histories; no significant differences were observed in mental component scores. Consistent with the findings of Okonkwo et al. [29], the difference in PCS scores between subjects with and without a stroke history in this study was approximately twofold that in MCS scores. In contrast to our findings on stroke effects, Haley et al. [30] indicated that an incident of stroke has a negative impact in both mental and physical health over a three-year period. One possible explanation for these different findings is that the elders in our present study had already coped with their conditions, allowing for better mental health.

In present study, we used Cohen’s d in the chi-square value formula ( , where N is total sample size) [31] to calculate the effect size of significant factors. Our results indicated a mid-size effect of both pain (d = 0.47) and frailty (d = 0.38). The effect sizes of other factors (including age, seeing relatives and friends at will, fall histories, and sleep disorders) were small, between 0.2 and 0.3. The two determinants of pain problems and frailty were found to have greater relative association with successful aging.

The present study has certain limitations that should be taken into account when interpreting the results. First, owing to the nature of the cross-sectional study, we cannot explore the possible causal relationships between the health conditions considered in this study and successful aging. Second, the findings of our work are not generalizable to rural elders because this study sample was a group of metropolitan elders. Finally, the prevalence of successful aging in urban elders may have been overestimated due to the exclusion of elders who were diagnosed with dementia, had cognitive impairment, and were unable complete the SF-36 questionnaire. Our study’s elders reflected the general global measures of physical and mental dysfunction, rather than those of specific disabilities or impairments [32].

## Conclusions

The following conclusions can be drawn from the present study: the higher proportion of successful aging in elders was found among those who were relatively younger, had social support, not frail, and had no pain, sleep impairment, and fall history. These findings revealed the importance of considering suitable intervention to improve the frailty, pain, and sleep problems among the elders. However, the causal relationships between these factors and successful aging will require further investigations.
